# Maintenance of High Phytoplankton Diversity in the Danubian Floodplain Lake over the Past Half-Century

**DOI:** 10.3390/plants13172393

**Published:** 2024-08-27

**Authors:** Melita Mihaljević, Dubravka Špoljarić Maronić, Filip Stević, Tanja Žuna Pfeiffer, Vanda Zahirović

**Affiliations:** Department of Biology, Josip Juraj Strossmayer University of Osijek, Ulica Cara Hadrijana 8/A, HR-31000 Osijek, Croatia; mmihaljevic@biologija.unios.hr (M.M.); fstevic@biologija.unios.hr (F.S.); tzuna@biologija.unios.hr (T.Ž.P.); vcvijanovic@biologija.unios.hr (V.Z.)

**Keywords:** phytoplankton, taxonomic diversity, anthropogenic impact, extreme events

## Abstract

Riverine floodplains are recognized as centers of biodiversity, but due to intense anthropogenic pressures, many active floodplains have disappeared during the last century. This research focuses on the long-term changes in phytoplankton diversity in the floodplain lake situated in the Kopački Rit (Croatia), one of the largest conserved floodplains in the Middle Danube. The recent dataset from 2003 to 2016 and historical data from the 1970s and 1980s indicate high phytoplankton diversity, summarising 680 taxa for nearly half a century. The variability of species richness is driven by specific in-lake variables, particularly water temperature, water depth, total nitrogen, pH, and transparency, determined by a redundancy analysis of the current data. The high phytoplankton diversity levels are sustained regardless of intense pressures on the lake environment, including exposure to strong anthropogenic pollution in the past and extreme hydrological events, both droughts and floods, which have increasingly affected this part of the Danube in the last decades. The conserved hydrological connection between various biotopes along the river–floodplain gradient seems crucial in maintaining high phytoplankton diversity. Accordingly, conserving natural flooding is mandatory to maintain high biodiversity in complex and dynamic river–floodplain systems.

## 1. Introduction

Riverine floodplains are dynamic spatial mosaics of running and standing waters, permanent and temporary waters, wetlands, and groundwater in which water plays a vital role in connecting various landscape patches [1]. This hydrological connectivity drives the highly complex multidimensional exchange pathways of water, dissolved and particulate matter, and biota between the river and the floodplain habitats at different spatial and temporal scales [2]. Therefore, riverine floodplains are recognized as highly productive systems and centers of biodiversity and bioproduction [3], so most groups of aquatic organisms show high species diversity and strong patterns of ecological dynamics in response to habitat heterogeneity and water level fluctuations [4]. Biodiversity hotspots are often found near rivers, banks, and floodplains, representing habitats with high structural and functional dynamics [5]. However, indicators for complex ecosystems such as floodplains, which are determined by parameters and processes that are difficult to measure directly (e.g., the frequency and duration of inundation), are of particular importance [6].

The problem of evaluating floodplain diversity arises because riverine floodplains have become the most threatened ecosystems worldwide due to continuous pressures. The hydraulic works of the last two centuries caused the fragmentation of floodplain habitats, and globally, most of the large active floodplains have disappeared. Quantifying multiple human pressures and their relationship with the ecological status of all European rivers found that better ecological status is associated with natural areas in floodplains [7]. Unfortunately, an overview of floodplain loss in Europe [8] showed that, depending on the river, 70–100% of the former floodplains had been lost over past centuries. Generally, the loss of habitats leads to decreased biodiversity and reduced ecosystem functions [9].

The situation is further exacerbated because the remaining floodplains have become particularly vulnerable to climate change due to their sensitivity to alterations in the hydrologic cycle. For floodplain lakes, the impact of hydrological changes on the lake’s ecology is perhaps much higher than other climatic factors [10].

Climate change models predict increased occurrences of extreme events (flooding, extended droughts), which will amplify the seasonal and multiannual amplitude of water level fluctuations, in turn creating hydrological stresses (e.g., prolonged hydraulic retention time) in aquatic habitats [11]. Numerous documents from actual research, summarized by [12,13], show that unprecedented changes in precipitation patterns have been recorded globally in recent decades, and further changes can be expected to occur shortly. This global loss of species in freshwater ecosystems, especially wetlands, is a direct consequence of the impact of climate change [14].

The common goal of biodiversity conservation following the Convention on Biological Diversity [15] has been recognized globally as one of the main priorities in environmental protection. Currently, diversity is one of the most frequently used quantitative descriptors of communities because diversity metrics can describe system properties such as complexity [16] and ecosystem functions [17,18].

Algae are usually characterized by high species richness and consist of relatively easy-to-identify taxa, making them suitable to answer various ecologically relevant questions [19]. A high diversity of phytoplankton is one of the most remarkable features of aquatic systems [20], and today, phytoplankton represents one of the ecological components to be addressed in assessing the ecological status of freshwater ecosystems [21]. Various environmental factors drive phytoplankton taxonomic diversity in freshwater ecosystems, such as productivity, lake size, habitat diversity, or regional processes [22]. Additionally, the top-down control from fish to phytoplankton via zooplankton significantly influences phytoplankton and represents the basis for the functioning of the lake ecosystem [23].

The analysis of phytoplankton data from about 1500 lakes in 20 European countries by [24] revealed that two-thirds of the species that dominate lakes during the summer are dominant across Europe. The authors suggest that, in addition to nutrient concentrations, other water chemistry variables, such as alkalinity and the content of humic substances, have an important role in determining the distribution of the dominant phytoplankton species in European lakes. However, floodplain habitats differ considerably from other freshwater ecosystems, particularly in the hydrological sense. Flooding patterns and dynamics, recognized as the most important ecological factors for phytoplankton diversity and dynamics in the complex river–floodplain systems [25,26,27], for some species and in some instances, can be stimulating or disturbance factors, depending on the time of occurrence and intensity [28].

The Danube is the second largest river in Europe, connecting many ecoregions with exceptionally high freshwater biodiversity in central and eastern Europe and, therefore, is an important migration corridor for organisms in the Danube River Basin [29]. Our research focuses on a floodplain lake, Lake Sakadaš, a part of one of the largest conserved floodplains in the middle section of the Danube River known as Nature Park Kopački Rit (Croatia). This floodplain has retained a strong hydrological connection with the river, which can be considered a near-native condition [30]. Dragica Gucunski conducted the first comprehensive investigation of phytoplankton, motivated by scientific curiosity in the 1970s [31], making a valuable contribution to the initial efforts to protect this floodplain.

This study aims to elucidate leading factors influencing the lake’s phytoplankton taxonomic diversity in the Kopački Rit floodplain. For this purpose, we focused on the year-to-year variation of phytoplankton diversity in a time series of 14 years (2003–2016). To discern trends in the changes in biodiversity over a half-century, we compared three significant periods with regard to the specific ecological conditions. Finally, we created a complete list of phytoplankton species registered in this floodplain lake during the last half century to focus attention on the exceptional biodiversity of floodplain habitats and the necessity for their protection.

## 2. Results

### 2.1. Environmental Characteristics

**Figure 1:**
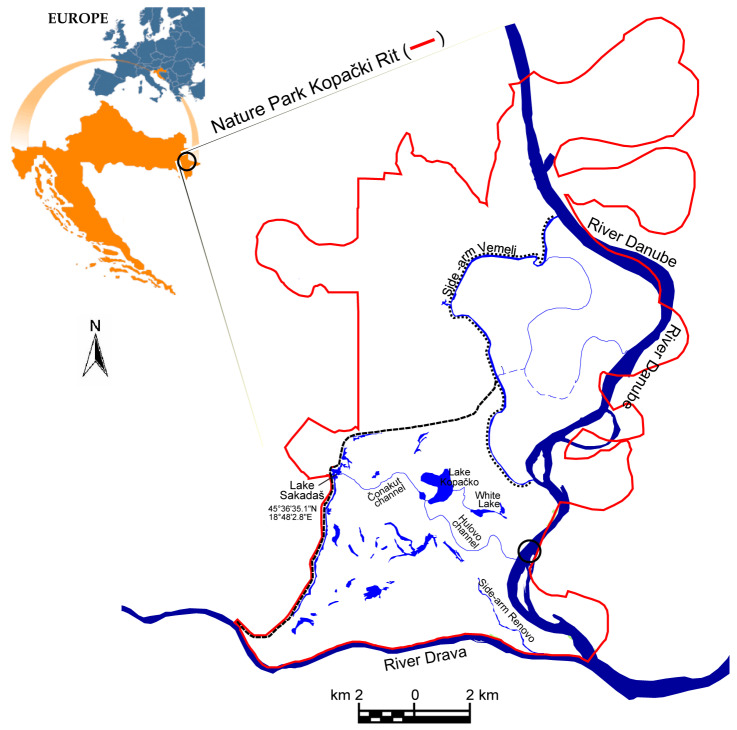
Study site—Danubian floodplain of Kopački Rit (Croatia).

The floodplain area of Kopački Rit (Figure 1) belongs to the Pannonian ecoregion, which is influenced by a continental climate. The analysis of meteorological and hydrological data collected during the past century [32] showed an increase in mean annual air temperature (11.49 °C) in the period 1988–2011 compared with the value (10.85 °C) obtained in the period 1900–1987. 

**Figure 2:**
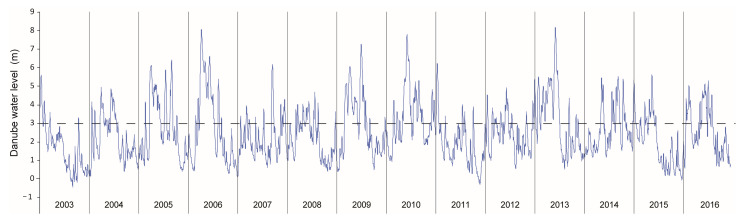
The daily oscillations of the Danube water level at the gauge station (river 1401.4 km) in 2003–2016. Note: flooding of Lake Sakadaš begins when the Danube water level rises above 3 m (dashed line).

The hydrological changes are reflected in the frequency of extreme hydrological conditions, both extreme flooding of the Danube and extreme dry periods in the floodplain, which happened more frequently in the past few decades [33], as was noted during the observed period 2003–2016 (Figure 2). Thus, extreme floods in the Middle Danube occurred in the spring and summer of 2006 and again in the summer of 2013, while the investigated floodplain area was affected by severe droughts during the summers of 2003 and 2015.

**Figure 3:**
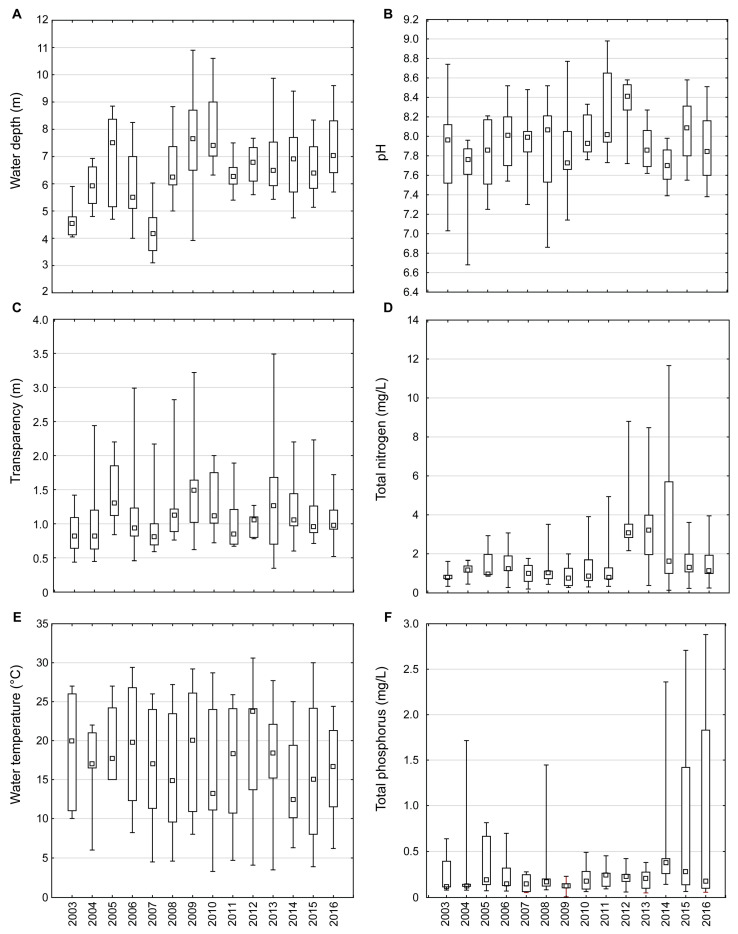
Interannual variability of physical and chemical water properties: (**A**) water depth; (**B**) transparency; (**C**) water temperature; (**D**) pH; (**E**) total nitrogen; (**F**) total phosphorus in Lake Sakadaš in 2003–2016. Central symbols represent medians, boxes represent the 25 and 75% quartiles, and whiskers represent minimum and maximum values.

**Figure 4:**
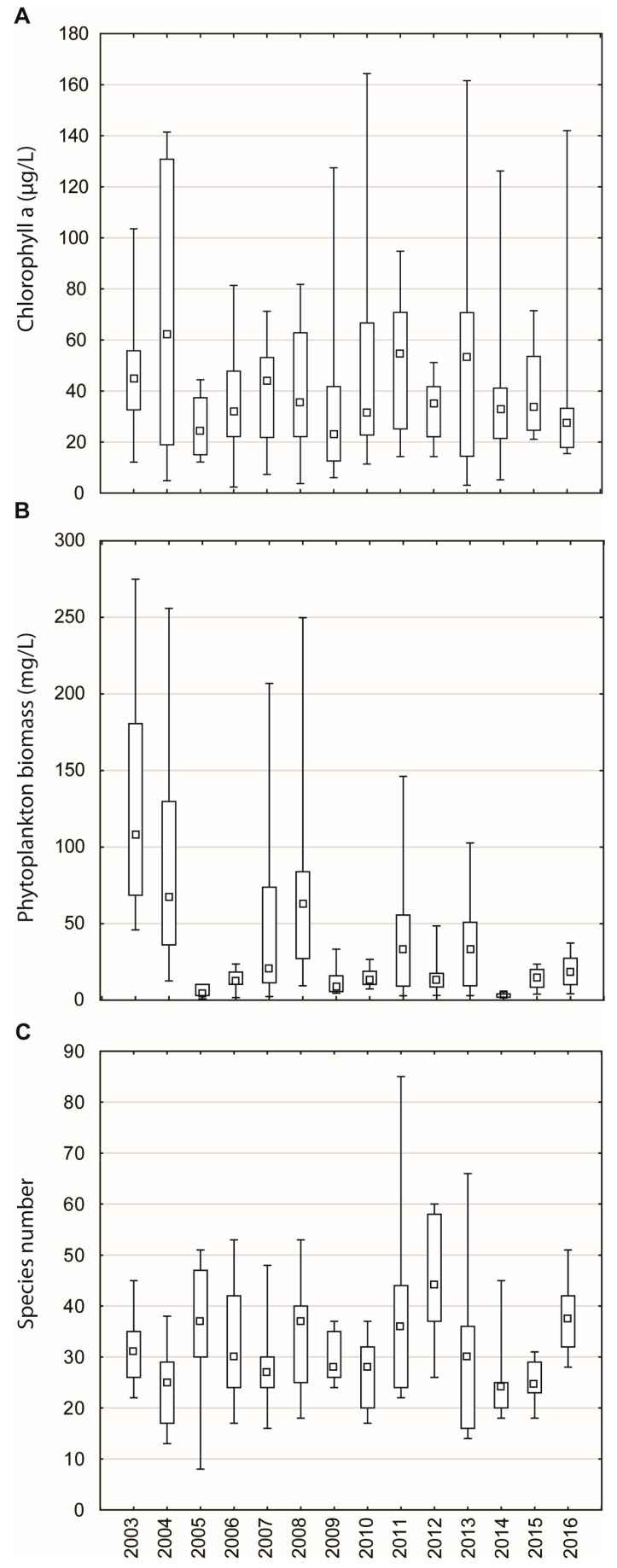
Interannual variability of chlorophyll-a concentration (**A**), total phytoplankton biomass (**B**), and species richness (**C**) in Lake Sakadaš in 2003–2016. Central symbols represent medians, boxes represent the 25 and 75% quartiles, and whiskers represent minimum and maximum values.

Depending on the changes in the water level of the Danube (Figure 2), i.e., the inflow of flood waters, the depth of the lake (WD) changed from 3.1 m to 10.90 m (Figure 3A). The significant oscillation of other physical and chemical water properties was as follows: transparency (SD) 0.3–3.5 m (Figure 3B); water temperature (WT) 3.3–30.6 °C (Figure 3C); pH 6.7–9.0, with significantly higher values in the period from 2010 (Figure 3D); total nitrogen (TN) 0.1–11.7 mg/L, with a substantial increase in 2012–2014 (Figure 3E); total phosphorus (TP) 0.01–2.88 mg/L, with a significant increase in 2014–2016 (Figure 3F), and large oscillations in chlorophyll-a (Chl-a) 2.34–164.37 μg/L (Figure 4A). The lake was in an eutrophic/hypertrophic state according to the OECD system [34].

### 2.2. Phytoplankton Diversity and Dominant Taxa

Half-century-long investigations of phytoplankton in Lake Sakadaš summarised 680 taxa belonging to 231 genera. Most species found in the phytoplankton samples are typically planktonic; however, several species of benthic, metaphytic, or periphytic origin are also included in the total biodiversity. The analysis of our recent data from 2003 to 2016 identified 28 dominant phytoplankton species, among which cyanobacteria and diatoms were the most represented, with 8 species (Table 1).

Generally, a great diversity of cyanobacteria was found (73 taxa) and sorted into 38 genera. Cyanobacterial species were continuously found all year around, blooming in warm water conditions, as expected. Still, infrequently abundant populations of particular species were registered in periods of very cold water during winter. The occasional appearance of chroococcalean bloom-forming species *Merismopedia* spp. and *Chroococcus* spp. has been recorded mainly in the past. However, during the entire period of observation, the most important bloom-forming species are filamentous cyanobacteria, mostly dinitrogen-fixing nostocalean species *Aphanizomenon flos-aquae*, *Dolichospermum circinale* (Rabenhorst ex Bornet and Flahault) Wacklin, Hoffmann and Komárek, *D. planctonicum* and *D. solitarium*, as well as non-N2-fixing species *Planktothrix agardhii*, *Limnothrix redekei*, *Pseudanabaena limnetica,* and *Planktolyngbya limnetica*.

Chlorophyta was the most diversified group, representing 99 genera and 274 taxa. As a group, coccal green algae (Chlorococcales) significantly contribute to the total phytoplankton abundance with large species numbers. Still, individually, none of those species are found to be mass-developed and have a high contribution to the total biomass. Species from the genera *Actinastrum*, *Crucigenia*, *Coelastrum*, *Desmodesmus*, *Kirchneriella*, *Monoraphidium*, *Pediastrum*, *Scenedesmus*, and *Tetraedron* are common in well-flushed warm waters during the late spring and summer months.

The high diversity of Bacillariophyceae (180 taxa) was found, and their appearance and domination in phytoplankton communities are strongly connected with mixing events during the flooding of the lake, which can happen at any time of the year. With the early spring flooding, there is a higher abundance of small centrics dominated by *Stephanodiscus hantzschii*. In contrast, later in spring, *Melosira varians* and pennates from the genera *Fragilaria*, *Navicula*, *Cymbella,* and *Gomphonema* contributed to the diversity of diatom assemblages. In the condition of higher water temperature and decreasing water mixing in the late spring–early summer period, *Aulacoseira granulata* was prominent.

Xanthophyceae comprised 21 taxa belonging to 9 genera (*Arachnochloris*, *Centritractus*, *Chlorobotrys*, *Chloropedia*, *Goniochloris*, *Ophiocytium*, *Pseudotetraedron*, *Tetraëdriella*, and *Tetraplektron*). Among 27 taxa belonging to 13 genera of Chrysophyceae, the species *Synura uvella* and *Dinobryon divergens* usually contribute significantly in the early spring and late autumn communities. An almost monodominant *S. uvella* bloom was noted in the past (September–October 1972) after extreme flooding and the inflow of floodwaters into the lake.

Euglenophyta comprised 9 genera represented by a high number of taxa (76), with the most diversified genus being *Trachelomonas* (22 taxa). Abundant populations of mixotrophic euglenophytes, a variety of species of genus *Euglena*, *Phacus*, *Lepocinclis,* and *Trachelomonas*, usually occur in conditions of low water and high content of organic matter due to the collapse of macrophyte vegetation in autumn.

Although the number of identified dinoflagellates (8 genera with 23 taxa) and cryptophytes (4 genera with 9 taxa) was relatively low compared to other groups, most of these species are very prominent in the seasonal dynamic of phytoplankton. Thus, dinoflagellates *Ceratium hirundinella* (O.F.Müller) Dujardin and *Peridinium* species make a significant component of phytoplankton during the “dry phase” in autumnal conditions when the lake is hydrologically isolated from flood waters. Seasonal timing of cryptophytes (*Cryptomonas* spp.) is mainly related to the late spring increase in water temperature and to the end of spring diatom maximum. At the same time, some species, like *Plagioselmis nannoplanctica*, are abundant throughout the year.

### 2.3. Interannual Variation of Biomass and Diversity

Total phytoplankton biomass varied significantly in the observed period (Figure 4B), with the cyclic shift between phytoplankton turbid state characterized by high biomass (up to 100–230 mg/L) and phytoplankton clear state characterized by low phytoplankton biomass (less than 50 mg/L). A turbid state was established in 2003–2004, 2007–2008, 2011 and 2013. Interannual variability in biomass—the lowest was in 2014: 0.01 mg/L (25 species)–5.38 mg/L (20 species) mg/L; the highest was in 2008: 9.40 mg/L (24 species)–249.82 mg/L (41 species).

The total number of phytoplankton species in a single sample ranged from 8 to 85, reflecting the lake’s rich biodiversity (Figure 4C). The interannual variability in species richness was the smallest in 2015, with a range of 17 to 32 species, when the lake was in a clear state. In contrast, the largest annual oscillation was observed in 2011, with a range of 22 to 85 species, when the lake was in a turbid state.

**Figure 5:**
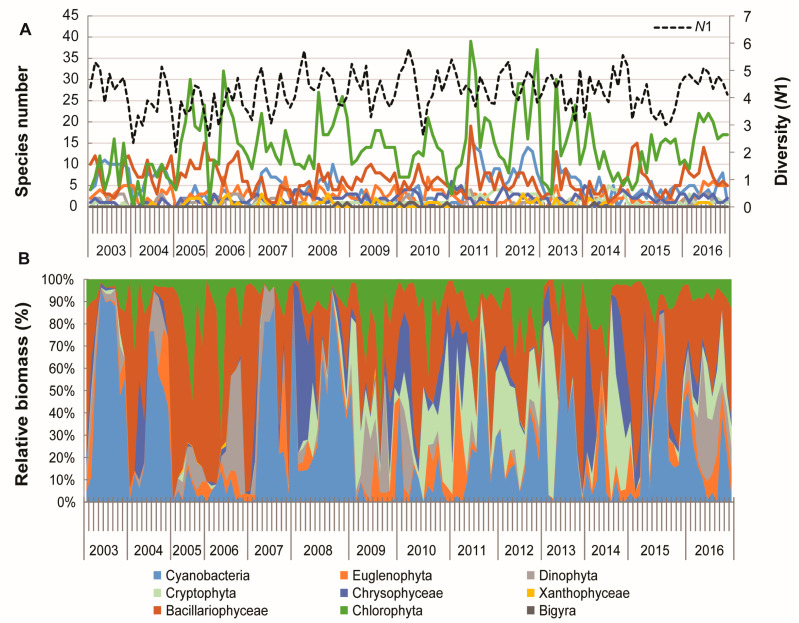
Interannual variation in (**A**) species number and diversity (*N*1) and (**B**) relative contribution of biomass of phytoplankton groups in Lake Sakadaš over a period of fourteen years (2003–2016).

The lowest diversity (*N*1 = 2.0) was found in March 2005 (Figure 5A) when a total of eight species (four species of Bacillariophyceae and four species of Chlorophyta) contributed more than 90% to the total biomass of only 0.81 mg/L (Figure 5B), with the domination of diatom *Lindavia comta*. The highest number of species was observed in July 2011 when 85 species (19 species of Bacillariophyceae (20% of total biomass); 16 species of Cyanobacteria—24% of total biomass; two species of Cryptophyta—22% of total biomass; 39 species of Chlorophyceae—19% of total biomass; 3 species of Euglenophyta, 4 species of Dinophyta, and 2 species of Chrysophyceae contributed to the total biomass of 56.0 mg/L.

In the condition of diatom blooming, as in March 2007, only two diatom species contributed 93% of the total biomass (19.78 mg/L), with a total phytoplankton diversity of 16 species. In the condition of cyanobacterial bloom, as in September 2008, cyanobacterial species reached 93% of total biomass, and 41 species contributed to total diversity (10 species of Cyanobacteria, 19 species of Chlorophyta, 4 species of Bacillariophyceae) (Figure 5A,B).

### 2.4. Similarity Indices

To illustrate the changes in interannual variability of phytoplankton during the long-lasting period, we used standard diversity indices and compared three phytoplankton species matrix of data obtained in the continuous year-to-year monitoring: (i) 1972/1973 [35], which represents the first comprehensive historical data of phytoplankton; (ii) 1977/78, when the lake was strongly impacted by pollution [36], and (iii) in 2007/2008, which represents the current state under the more or less usual hydrological condition. The given results (Table 2) reflected wider variations in the taxonomic composition of phytoplankton.

The similarity indices [37,38] showed higher similarity between historical data (periods 1972/73 and 1977/78) than when comparing these two periods with more recent data (period 2007/2008). The lowest diversity (*N*1 = 3.14) was established in 1977/1978 after the strong anthropogenic pressure on the lake environment by direct wastewater inflow from agricultural farms. Therefore, many of the Bacillariophyceae taxa, as well as some Cyanobacteria and Chrysophyceae taxa, which were characteristic representatives during the initial investigations of the lake, disappeared in the phytoplankton community. The development of the monotypic community of *S. hantzschii* (November 1977) was followed by a continuous decrease in the number of phytoplankton taxa. More recent data (2007/2008) also show lower diversity (*N*1 = 3.24) when compared to initial investigations (*N*1 = 3.36), which is mainly related to the lower number of Bacillariophyceae species. Moreover, significant changes are seen in the species composition within the main phytoplankton groups, especially Cyanobacteria, where the number of chroococcal species declined at the expense of filamentous taxa. Also, recent data show a significantly higher diversity of chlorophytes than previous data.

### 2.5. Ordination

**Figure 6:**
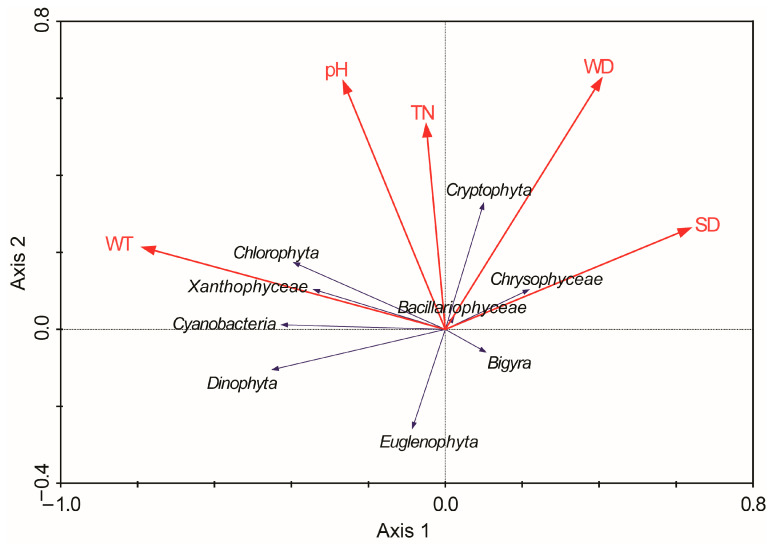
RDA biplot based on phytoplankton group (Bacillariophyceae, Bigyra, Chlorophyta, Chrysophyceae, Cryptophyta, Cyanobacteria, Dinophyta, Euglenophyta, Xanthophyceae) richness and the significant environmental variables (*p* < 0.05) for 14 years (2003–2016) in Lake Sakadaš.

The RDA analysis yielded significant insights into the influence of environmental variables on phytoplankton richness in Lake Sakadaš for the 14-year period (2003–2016) (Figure 6). The eigenvalues for the first two main RDA axes were 0.098 and 0.029, explaining 22.5% of the total variance of species data. The first ordination RDA axis was strongly correlated with TN and pH, while the second ordination RDA axis was strongly correlated with WT and SD. The results of the Monte Carlo test showed that WT (F-ratio = 9.67, *p* = 0.002) was the main environmental factor explaining the variations in phytoplankton richness, followed by WD, TN, pH, and SD. The richness of Chlorophyta, Xanthophyceae, Cyanobacteria, Dynophyta, and Euglenophyta was positively correlated with WT, pH, and TN, while the increased WD and SD contributed to the development of Cryptophyta, Bacillariophyceae, and Chrysophyceae.

## 3. Discussion

### 3.1. Total Diversity

A total of 680 taxa registered in the investigated floodplain lake during almost half a century is an impressive phytoplankton diversity. It is difficult to compare this number with the diversity of floodplain lakes on a European scale. Above all, it is because most of the large floodplains disappeared in the past centuries. Additionally, there is a lack of data from long-term monitoring of the remaining floodplains. However, comparing different floodplain habitats along the Danube River showed that phytoplankton diversity varies significantly between water biotopes along the river–floodplain systems. Thus, rich algal flora characterizes floodplain lakes [39,40], compared with substantially lower diversity in the sidearms and temporary wetlands [41].

Our research has shown that the species richness in the floodplain lakes we studied is comparable to that of subtropical and tropical areas still in near-pristine conditions. For instance, the species diversity of 397 taxa found in a long-term (2000–2013) phytoplankton investigation of a subtropical floodplain lake directly connected to the Paraná River [42] is comparable to the total of 344 species we determined in the investigated lake over the period 2003–2016. Furthermore, the phytoplankton alpha diversity per sample in the Upper Paraná River floodplain, ranging between 4 and 87 species with a mean of 30 (±16.5) species [43], aligns with the variation (8 and 85 species (±31.85)) we observed in the investigated lake.

### 3.2. Diversity Drivers

The rich diversity of phytoplankton in the floodplain lakes can be attributed to a complex interplay of local and regional processes. Local processes, such as environmental variables operating on the in-lake level, and regional processes, including dispersal, are known to structure microbial communities [44]. Our RDA analysis further revealed that specific in-lake variables, including WT, WD, TN, pH, and SD, significantly influenced alpha phytoplankton diversity in the studied lake. The water level fluctuations, a result of changes in hydrological regimes, can profoundly impact underwater light availability, mixing regime, nutrient cycling processes, lake volume, and extent of the littoral zone, with significant implications for phytoplankton dynamics [45], particularly diatoms, as demonstrated by the RDA analysis.

Nutrient status, i.e., the eutrophic character of the lake, to a certain respect, is responsible for the high diversity [46] as well as for the high abundance of particular species. Despite a high level of nutrients in the water, almost always enough for the unlimited development of many species, the lake still undergoes successive changes of phytoplankton clear and turbid state. A turbid state implies the appearance of a long-lasting bloom of, most often, cyanobacterial species. Interestingly, the total phytoplankton diversity remains relatively high, even when only two or three cyanobacterial species contributed more than 80% of the exceptionally high total biomass. Similar values of biodiversity were also determined in the conditions when the lake was in a clear state. Altogether, such sustainability of phytoplankton diversity can be linked to the preserved natural dynamics of flooding. As is well known, the longitudinal flow of the main channel and its hydrological fluctuations in a floodplain system that connect and isolate the environments directly influence lateral and longitudinal exchanges, dilution, and the dispersion and colonization of phytoplankton species [42]. The studied floodplain is characterized by many habitats, including permanent marshes/pools, ponds, and swamps on organic soils with emergent vegetation, shallow lakes, and river sidearms, interconnected during the flooding period. Therefore, spatial structuring of the habitats and the high level of environmental complexity can significantly contribute to maintaining high phytoplankton diversity [47]. This has also been proven in the small stratified oxbow lake [22] and the lakes in a large complex Amazonian floodplain [48].

Floodwaters may influence the floodplain biotope assemblages by serving as a source of colonizers that develop in the lake during and after flooding or by flushing out wetland algae or changing environmental conditions [49]. Water mixing caused by flood turbulence is a favorable environment for many dominant diatom species in the investigated lake. Thus, the presence of *M. varians* and certain species of the genus *Fragilaria*, *Navicula*, *Cymbella,* and *Gomphonema*, species characteristic of highly lotic environments [50], is connected with strong mixing events during the flooding phase [51,52]. Additionally, flooding supports the re-suspension of meroplanktonic and benthic algae in the water column. Many species of *Aulacoseira* and *Stephanodiscus* are meroplanktonic, while *M. varians* and *Navicula cryptocephala* Kützing are typical benthic species, and they can all be regularly found in the plankton when the water column undergoes turbulent mixing [51]. Therefore, the lake sediment offers additional niche space and thus can contribute to higher phytoplankton diversity [53].

Our previous research revealed that the timing and magnitude of flooding could be crucial for the abundance of many species [54]. For example, the sustainability of cyanobacteria *A. flos-aquae* and *Dolichospermum* spp., which are particularly sensitive to water mixing [50,55], was closely connected to hydrological stability during the isolation period in the summer, while turbid and mixed waters, with intensive interaction between sediment and water, favored the dominance of *P. agardhii* [56].

Floods are favorable for diatoms in several ways. First of all, there are exchanges of tychoplanktonic taxa, particularly diatoms, along a river–floodplain gradient, but due to the intensive interactions, it is not possible to clearly observe the rate of species input from the river to the floodplain or vice-versa [57]. For example, with the early spring flooding, there is a higher abundance of small centrics dominated by *S. hantzschii*, which coincides with its early bloom in the Danube [54].

### 3.3. Diversity Stressor—Anthropogenic Impact

Any change generated by natural stress and anthropogenic impacts that alter lakes’ physical and chemical processes can affect the phytoplankton community to react rapidly to environmental modifications at different time scales [45]. Different stressors such as catchment disturbance, pollution, water resource development, and biotic factors (e.g., invasive species) affect water quality and aquatic biodiversity [58]. Our results show that the strongest anthropogenic impact was in the 1970s when wastewater from nearby pig farms was directly discharged into the lake. In river-connected floodplain lakes, such as this lake, the amount of sewage input and water nutrients to lakes influenced by the hydrological conditions can result in significant lake degradation [59]. Among the few measured physical and chemical factors of water properties originating from that time, extremely high pH values and frequent occurrence of anoxia throughout the water column [35] indicate a dramatically deteriorated lake environment. Hypertrophic conditions often lead to monotypic blooms, resulting in low-diversity and high-biomass phytoplankton assemblages [60]. A heavy bloom of green algae (*Eudorina*, *Pandorina*, *Pleodorina*) appeared in July 1973 in highly alkaline conditions (pH 9.7), and another heavy bloom of cyanobacteria *Spirulina* sp. occurred after that, confirming remarkably bad ecological conditions in the lake. Moreover, this represents an extreme environment for phytoplankton similar to alkaline lakes or soda lakes, which are characterized by extremely high salinity values and the almost monodominance of *Spirulina* [61]. This demonstrates that land use types reflecting anthropogenic pressures could be critical drivers explaining phytoplankton structure in river-connected lakes [62]. In addition, a potential linkage between eutrophication and pollution-mediated loss of phytoplankton is expected [63]. Therefore, a continuous decline in the number of species was established during the uncontrolled anthropogenic impact, from 231 species in 1970 [64] to 144 species in 1972/73 [35] and only 96 species in 1977 [36].

Thanks to the cessation of the direct inflow of wastewater into the lake, the lake’s biodiversity has recovered, as indicated by comparing three characteristic periods by the similarity indices. Significantly higher diversity of chlorophytes in more recent datasets than historical ones can indicate the eutrophication tendency [65] but as a result of natural processes rather than direct anthropogenic influences.

### 3.4. Herbivore Pressure

Herbivore predation can affect phytoplankton by reducing biomass, altering taxonomic composition, and shifting the dominance of different species [66,67].

Dissimilar life features of the most prominent zooplankton groups in Lake Sakadaš that have a substantial herbivorous component in their diet, i.e., ciliates, rotifers, cladocerans, and copepods, enable them to respond differently to changes in environmental characteristics in these hydrologically variable ecosystems. Different waterbodies within a floodplain area of Kopački Rit support different zooplankton assemblies that highly depend on species-specific adaptation responses to hydrology [68]. Fluctuations in water level and abiotic components influence shifts in zooplankton assemblages that alter levels of secondary production (top-down) and herbivory ratios (bottom-up) [69]. The most numerous zooplankton group, rotifers, show the most active response to the hydro regime change, while copepods discriminate different habitat types in different hydrological phases. Rotifer feeding types and grazing intensity reflect variations in hydrological conditions due to food availability [70]. Zooplankton food selectivity also has a substantial impact on plankton dynamics. For example, copepods usually suppress large phytoplankton, while cladocerans frequently control small algal groups [71]. Although zooplankton causes a decrease in algal density, herbivory can enhance the phytoplankton functional diversity and decrease the control of stronger competitive species that are grazer-resistant [72].

### 3.5. Invasive Species

Invasive alien species are recognized as one of the most critical indicators of threats to biodiversity. Their number has continued to rise in the past decades in all types of ecosystems in the pan-European area [7]. The Danube is part of the Southern Invasive Corridor, which links the Black Sea with the North Sea, and it is one of Europe’s four most important routes for invasive species [29]. Therefore, the Danube Basin is exposed to intensive colonization and further spreading of invasive species [73].

Among hundreds of phytoplankton species in the investigated lake, several species can be classified as alien and potentially expansive in European waters [74]. Some have frequently appeared in the lake, but their expansive development has not been recorded, such as chlorophytes *Monactinus simplex* (Meyen) Corda and *Staurastrum pingue* var. *planctonicum* (Teiling) Coesel and Meesters. Some potentially expansive nostocalean cyanobacteria appear only sporadically, such as *Cuspidothrix issatschenkoi* (Usachev) P.Rajaniemi, Komárek, R.Willame, P. Hrouzek, K.Kastovská, L.Hoffmann and K.Sivonen and *Dolichospermum compactum* (Nygaard) P.Wacklin, L.Hoffmann and J.Komárek. However, few potentially toxic tropical–subtropical species have had and are still experiencing significant expansion in the floodplain waters. In the past, a heavy bloom of *Raphidiopsis mediterranea* Skuja was registered in the summer of 1973 [35], but there are no records of its massive development after that finding. However, the bloom of *Raphidiopsis raciborskii* (Woloszynska) Aguilera et al. has appeared more frequently in recent decades. *R. raciborskii* is a global invasive species [75] whose spreading along the various freshwater habitats in the Danube Basin started between 1975 and 1980 [76]. Its bloom has been registered in various freshwater habitats along the Lower Danube [77] and the Upper Danube [78]. The first finding of *R. raciborskii* bloom in Lake Sakadaš was in the summer of the extremely dry year 2003 when its over-domination in the total biomass of phytoplankton longer than two months led to establishing an equilibrium phase [28]. Afterward, a bloom of *R. raciborskii* occurred periodically, first in the summer of 2004 [28], in the late spring and summer of 2007 [56], in the summer of 2013 and 2015, and in the early autumn of 2016 [79]. It is known that *Raphidiopsis* appears to be associated with longer water residence times [80]; therefore, the lack of its development in specific years can be related to the highly variable hydrological conditions.

Among diatoms, *Skeletonema potamos* (C.I. Weber) Hasle, one of the most common phytoplankton species in the Danube River in its middle section, is a potentially invasive species in the floodplain waters [81]. With relatively low abundance, *S. potamos* was frequently found in the floodplain lake during the observed period. However, its populations were more abundant in the main river bed of the Danube during the conditions of high water temperatures and low water levels in summer–autumn periods [52]. A substantial increase in *S. potamos* abundance in the Hungarian stretch of the Danube from 1979 to 2012 correlated with increasing water temperature over the same period [82]. Based on those findings, authors predict possible expansion of its geographic range and increase in seasonal duration within existing habitats in response to the warming of surface waters.

### 3.6. Global Change

There is growing evidence that climate change will significantly alter ecologically important attributes of hydrologic regimes in rivers [83], among which are the dynamics and intensity of floods that are particularly significant for floodplain habitats. Extreme hydrological events on the Danube along the investigated floodplain, both extreme flooding and dry periods, have been documented in the observed period. Thus, the heat wave of 2003 produced long-lasting dry conditions in the floodplain suitable for prolonged heavy cyanobacterial bloom dominated by alien tropical species. However, there was no significant decline in diversity as might be expected with increased cyanobacterial biomass, as was, for example, noted in the small shallow eutrophic lake [84] and also in a peri-alpine lake in the extremely hot summer of 2003 [85]. Although cyanobacterial blooms alter dominance relations in the phytoplankton and reduce light availability in waters, it does not necessarily coincide with eliminating other species and reducing their species numbers or diversity [86]. The sustainability of phytoplankton diversity was also shown in the extreme flood conditions in 2006 and 2013, when total biomass declined without a decrease in total phytoplankton diversity. As was emphasized [87], even if the highest diversity is reached at intermediate levels of disturbance, intensive floods (which promote high connectivity) are essential for the exchange of propagules, nutrients, and organisms among habitats, and such exchanges during high waters are mandatory to keep high biodiversity unique to river–floodplain systems because the exchanges increase the probability of rare species to disperse and colonize new sites during floods. Nevertheless, hydrological analyses of the Danube at the river’s stretch along the investigated floodplain [33] showed that the probabilities of more extended flooding periods occurring have decreased alarmingly in the last few decades. After all that has been presented so far, it is clear that this could have a significant negative impact on total biodiversity, including phytoplankton.

## 4. Materials and Methods

### 4.1. Study Site

The Kopački Rit floodplain is an inundation area of the Danube along the river 1410–1383 km (Figure 1). This floodplain was formed as a result of a specific combination of geological and hydrological conditions that created a complex delta-shaped relief (confluence of the Drava River into the Danube River), filled with fluvial and marshy sediments and, therefore, represents a distinctive feature on the European level [88]. In this river section, the Danube is a typical lowland river with a mean annual discharge of 2085 m^3^ s^−1^ and a yearly mean water level of 2.63 m (data source—daily recordings in the period 1987–2008 at the gauge station at river 1401.4 km). Despite the flatland sight, in the complex microrelief pattern of the floodplain, shallow lakes and ponds are significant, as well as the network of natural channels.

Lake Sakadaš is the deepest water depression located in the western part of the floodplain, and it is through two channels (total length ca. 10 km) in a direct hydrological connection with the incoming floodwaters from the main river channel of the Danube. The flooding pattern depends on the fluctuations of the Danube water level and is highly variable between years. Floods may occur at any time of the year, but the most frequent floods arise in spring and early summer. The lake’s average depth during the isolation from the riverine waters is usually about 4–5 m, with a surface of about 0.15 km^2^, while during high-intensity flooding, the lake depth can reach more than 8 m [88].

In the past, e.g., during the 1970s, the income of wastewater from the surrounding agricultural area caused a dramatic increase in the eutrophication of the floodplain waters. A gradual deterioration of water quality as a consequence of organic pollution was expressed as an increase in pH (up to 10) and an increase in suspended matter and nutrients [36]. Frequent, long-lasting anoxic conditions occurred during that time, causing massive fish mortality [89]. A remarkable improvement in water quality occurred in 1985 due to the permanent cessation of wastewater discharges into the lake and the strong protection of the larger floodplain area with the status of a Nature Park. Moreover, sediment removal from the whole lake bottom applied in 1984 proved to be a successful measure for the lake revitalization, resulting in a significant decrease in nitrate and total nitrogen contents [88]. After that, hypoxic conditions were restricted to the water layer near the bottom and appeared only occasionally at the end of the vegetation season due to the decomposition of organic matter from natural accumulation. However, a natural process of eutrophication that occurred after that was expected and primarily is associated with dynamic patterns of matter and nutrients along the river–floodplain gradient.

The floodplain of Kopački Rit is one of the main spawning, feeding, and refuge hotspots for fish native to the Danube Basin. The ichthyofauna is rich and diverse, comprising about 50 species [90], among which the most abundant species are silver bream (*Blicca bjoerkna* Linnaeus), Prussian carp (*Carassius gibelio* Bloch), and bleak (*Alburnus alburnus* Linnaeus), while two invasive species, *C. gibelio* and silver carp (*Hypophthalmichthys molitrix* Valenciennes), dominate in biomass [91].

The Kopački Rit floodplain is internationally recognized as a Wetland of International Importance (Ramsar site), a part of the Natura 2000 network, and since 2021, has been a part of the world’s first 5-country UNESCO Biosphere Reserve Mura—Drava—Danube (TBR MDD).

### 4.2. Sampling and Field Analyses

Sampling was performed monthly from 2003 to 2016, mandatory from March to November, and depending on the weather and hydrological conditions in the remaining months, totaling 135 samples in this study. Surface water samples were collected at the sampling station in the central part of Lake Sakadaš. Selected water quality parameters (temperature (WT), pH, conductivity (Cond), and dissolved oxygen (DO)) were measured on-site in the subsurface (20 cm) using a WTW Multi 340i portable meter (Wissenschaftlich-Technische Werkstätten, Germany). Water transparency (SD) was estimated using a Secchi disc, and water depth (WD) was measured using a weighted rope. Concurrent samples were taken for Chl-a and nutrient analyses. For the qualitative analysis of phytoplankton, 10 L of subsurface water was collected from the lake, filtered through a 25 μm mesh phytoplankton net, and preserved in 4% formaldehyde. Unfiltered phytoplankton samples were collected and fixed in acidified Lugol’s solution for quantitative analysis.

### 4.3. Laboratory Analyses

For Chl-a determination, water samples were filtered through Whatman GF/C glass fiber filters (Whatman International Ltd., Maidstone, UK), and pigments were extracted in acetone. Absorbance was measured spectrophotometrically, and the Chl-a concentration was calculated [92,93].

Samples were analyzed for nutrient content according to [94] and standard methods for ammonium (NH4-N) [95], nitrates (NO3-N) [96], nitrites (NO2-N) [97], Kjeldahl (orgN) [98] and total nitrogen (TN) [99], and total phosphorus (TP) [100].

Phytoplankton taxa were identified by light microscopic observations to the lowest possible taxonomic level using the standard literature for species determination [101,102,103,104,105,106,107]. The nomenclature was updated according to the Algaebase website [108]. For the detailed diatom analysis, samples were subsequently cleaned in distilled water, treated with H_2_O_2_ and HCl, washed, and embedded on glass slides in Canada balsam or Naphrax. The Utermöhl [109] method was performed to quantitatively assess phytoplankton using an inverted microscope (Axiovert, Carl Zeiss, Inc., Jena, Germany). Phytoplankton biomass was estimated [110,111,112] and expressed as milligrams per liter fresh mass (mg/L).

### 4.4. Data Analyses

This research focuses on the alpha diversity of phytoplankton, which corresponds to within-community diversity and is measured as the species number within a floodplain lake [113]. To measure the compositional similarity of phytoplankton in different periods of investigation (1972/1973, 1977/1978, and 2007/2008), we calculated Sørensen’s similarity coefficient [37]:

$$S_{s}=\frac{2c}{a+b}$$

and the Jaccard’s similarity coefficient [38]:

$$S_{j}=\frac{c}{a+b+c}$$

These coefficients are based on the presence/absence of species, where *a* and *b* stand for the total number of species in two compared communities, respectively, and *c* stands for the number of shared species. To compare the changes in phytoplankton species diversity between the same three periods, we used Hill’s diversity number one (*N*1) [114], which measures the effective number of species in a sample and indicates the number of abundant species. It is calculated as the exponentiated form of Shannon’s diversity index (*H*′) [115], where

$$N1=exp(H^{’})$$

Detrended Correspondence Analysis (DCA) was used on phytoplankton richness data for the 14-year period (2003–2016) to determine whether the linear or unimodal statistical technique would be most appropriate for predicting responses. Since the value of the longest gradient did not exceed 3.0 [116], the linear method (redundancy analysis (RDA)) was used to assess the relationship between the phytoplankton richness and the measured environmental variables [117]. The phytoplankton richness data were log-transformed to meet the normality requirements. The following environmental variables were considered in the analysis: WL (mean values of the Danube water level for ten days before sampling), WD, SD, and the surface values of WT, DO, pH, NH4-N, NO2-N, NO3-N, TN, and TP. The forward selection option using Monte Carlo unrestricted test involving 499 permutations identified the significant subset of environmental variables, retaining only variables significantly (*p* < 0.05) contributing to the total variance of each species. DCA and RDA were performed with the CANOCO for Windows version 4.5 (Biometrics-Plant Research International, Wageningen, The Netherlands). A signed rank test was applied to analyze the differences in physical and chemical parameters, phytoplankton richness, and biomass using Statistica 14.0 (TIBCO Software Inc., Palo Alto, CA, USA).

## 5. Conclusions

Long-lasting observational data demonstrating a very high taxonomic diversity of phytoplankton in the investigated floodplain lake show that more than six hundred species have been registered during the past half-century. As a typical near-natural habitat in the complex river–floodplain gradient, the lake presents an opportunity for phytoplankton development due to the direct hydrological connection with diverse aquatic habitats that may accommodate numerous species. Consistency in phytoplankton diversity was established in clear and turbid phytoplankton states and conditions of extreme hydrological events. The highest stressor for phytoplankton diversity was the anthropogenic impact of organic pollution of waters in the past. At the same time, in the future, changes in the flooding dynamics as a consequence of global change seem to be essential for the persistence of high species richness and, therefore, the ecological stability of the floodplain lake.

## Figures and Tables

**Table 1:** List of dominant phytoplankton species contributing more than 25% to the total phytoplankton biomass recorded in Lake Sakadaš over the period 2003–2016.

Taxonomic Group	Species
Cyanobacteria	*Aphanizomenon flos-aquae* Ralfs ex Bornet and Flahault
	*Dolichospermum planctonicum* (Brunnthaler) Wacklin, L.Hoffmann and Komárek
	*Dolichospermum solitarium* (Klebahn) P.Wacklin, L.Hoffmann and J.Komárek
	*Limnothrix redekei* (Goor) Meffert
	*Planktolyngbya limnetica* (Lemmermann) J.Komárková-Legnerová and G.Cronberg
	*Planktothrix agardhii* (Gomont) Anagnostidis and Komárek
	*Pseudanabaena limnetica* (Lemmermann) Komárek
	*Raphidiopsis raciborskii* (Wołoszyńska) Aguilera et al.
Euglenophyta	*Trachelomonas oblonga* Lemmermann
	*Trachelomonas volvocina* (Ehrenberg) Ehrenberg
Dinophyta	*Gymnodinium* sp.
	*Peridinium aciculiferum* Lemmermann
Cryptophyta	*Cryptomonas erosa* Ehrenberg
	*Cryptomonas ovata* Ehrenberg
	*Cryptomonas* sp.
	*Plagioselmis nannoplanctica* (Skuja) G.Novarino, I.A.N.Lucas and Morrall
Chrysophyceae	*Chrysococcus rufescens* Klebs
	*Dinobryon divergens* O.E.Imhof
	*Synura uvella* Ehrenberg
Bacillariophyceae	*Aulacoseira granulata* (Ehrenberg) Simonsen
	*Aulacoseira* sp.
	*Lindavia comta* (Kützing) T.Nakov et al.
	*Melosira varians* C.Agardh
	*Stephanocyclus meneghinianus* (Kützing) Kulikovskiy, Genkal and Kociolek
	*Stephanodiscus hantzschii* Grunow
	*Ulnaria acus* (Kützing) Aboal
	*Ulnaria ulna* (Nitzsch) Compère
Chlorophyta	*Pandorina morum* (O.F.Müller) Bory

**Table 2:** Diversity indices used for comparing three phytoplankton species matrices (observed period 1972–2008) of Lake Sakadaš.

	**1972/1973–1977/1978**	**1977/1978–2007/2008**	**1972/1973–2007/2008**
Sørensen’s similarity coefficient (*S_s_*; %)	46.31	34.19	39.71
Jaccard’s similarity coefficient (*S_j_*; %)	30.13	20.62	24.78
	**1972/1973**	**1977/1978**	**2007/2008**
Hill’s diversity number one (*N*1)	3.36	3.14	3.24

## Data Availability

Raw data that support the outcomes of this study are available from the corresponding authors upon reasonable request.
